# Managing Hepatic Cysts in the Setting of Surgery for Cholecystitis

**DOI:** 10.7759/cureus.81054

**Published:** 2025-03-23

**Authors:** Patrick Dahdouh, Sarah M Gilyard, Yih-Dar Nien, Michael W Robinson

**Affiliations:** 1 Kinesiology, California State University, Northridge, USA; 2 Surgery, University of California Los Angeles David Geffen School of Medicine, Los Angeles, USA

**Keywords:** acute cholecystitis, cholelithiasis, chronic cholecystitis, hepatic cyst, laparoscopic cholecystectomy, right upper quadrant pain, surgery

## Abstract

Hepatic cysts are relatively common and often present with non-specific symptoms such as nausea and abdominal pain. While most are benign simple cysts, the differential diagnosis includes infectious, inflammatory, and malignant etiologies. Imaging, including ultrasound and cross-sectional modalities, is key to diagnosis. Hepatic cysts can occasionally present with symptoms that overlap with gallbladder disease, complicating the clinical picture.

We present the case of a patient with chronic cholecystitis who was found to have an incidental hepatic cyst obstructing the gallbladder. Given its size and obstructive nature, concurrent laparoscopic cholecystectomy and cyst fenestration with excision were performed. The procedure was completed safely, and the patient had an uneventful recovery. Histopathology confirmed a benign simple cyst. This case highlights the importance of recognizing hepatic cysts preoperatively and managing them intraoperatively to ensure safe surgical outcomes.

## Introduction

Cholecystitis is an inflammation of the gallbladder, a pyriform-shaped organ located in the right upper quadrant, inferior to the liver. The pathology of cholecystitis is classified as acute or chronic. Many explanations can underlie the pathophysiology of acute cholecystitis (AC). An infection, occlusion to the cystic duct, or compromise to the release of bile are among the common etiologies for acute cholecystitis [1-3]. Chronic cholecystitis is a result of AC symptoms, such as pain and nausea, sustained over a prolonged duration. Methodologies such as computed tomography (CT), hepatobiliary iminodiacetic acid (HIDA) scan, Murphy’s sign (with or without sonography), and ultrasound will present a variety of findings [1-3]. Thickening of the gallbladder wall, cholelithiasis, and the presence of pericholecystic fluid are sensitive signs of cholecystitis [2,4]

While attempting to identify and characterize the disease process, other conditions are occasionally identified that may confound the treatment of the gallbladder. In this case, the presence of cystic lesions in the liver poses a potential change to the overall treatment of the gallbladder*.* We will give an overview of treatment options, a unique case presentation, and factors to consider when treating concurrent gallbladder and hepatic cyst disease.

Hepatic lesions are diagnosed in an estimated 15% to 18% of the United States population [5,6]. These lesions present themselves in various ways, seen through diagnostic imaging methods. Methods such as ultrasound (US), computed tomography (CT), and magnetic resonance imaging (MRI) highlight these lesions [7]. 

Hepatic cystic lesions can be classified as infectious or non-infectious [5]. The cysts can be labeled across categories such as parasitic, non-parasitic, pre-malignant, malignant, benign, and traumatic [6]. With the complexity of hepatic lesions, the aforementioned imaging modalities are crucial to elucidate the origin and guide the treatment. Historically, imaging has not been accessible for diagnosing this condition. Hepatic cysts were often discovered through surgery in the mid-twentieth century [6]. Approximately 17 out of 10,000 individuals were diagnosed in the study [6]. The number of individuals worldwide currently diagnosed with hepatic cysts ranges between 5% to 10% [5]. Risk factors such as age and sex predispose patients to the development of cysts; in the classification of a simple cyst, there is a higher prevalence noted in females than in males [5]. These lesions may result as a congenital or chronic condition. Further, those in their 40s through 70s are more predisposed to liver lesions [6,8]. Some individuals in their 30s have developed symptoms and have required surgical intervention [8].

Hepatic cysts may impose little to no hindrance to a patient's life, as they generally are asymptomatic [9]. However, hepatic cysts can manifest in certain ways. Akin to cholecystitis, a patient may experience right upper quadrant (RUQ) pain, nausea, and shortness of breath [5].

The most frequent form of liver lesions is that of a simple cyst. Sizes can range from subcentimeter to over 30 cm. Hypotheses for the development of hepatic cysts include abnormality within a bile duct, which gradually dilates over time, in the case of polycystic liver disease (PCLD) [5]. While rare, cysts can rupture, causing pain and potentially intra-abdominal hemorrhage. Management for these cystic liver conditions varies depending on the type of hepatic cyst. For instance, a simple cyst may allow for surveillance, especially if deemed stable with progressive imaging. A ruptured, hemorrhagic cyst causing peritonitis should be managed surgically. 

With the other categories listed, the most common premalignant cyst is biliary cystadenoma (BCA), a slow, progressive condition branching from the bile ducts [5]. While there is controversy regarding the etiology of BCA, the condition is predominantly found in females [6]. Furthermore, the incidence of transformation from pre-malignancy to malignancy in the form of cystadenocarcinoma can range up to approximately 30% [5].

Additional hepatic lesions such as biloma and cystic liver metastases are a result of a variety of pathophysiology. A biloma - a capsule filled with hepatic bile collection - can be a result of a cholecystectomy or traumatic liver injury [6]. This condition can generally resolve with percutaneous drainage, as is the case with infected bilomas. Cystic liver metastases may arise across numerous structures within the body (i.e., renal and ovarian malignancies) and connote a poorer prognosis. It is imperative to identify and formulate a treatment plan. Treatment options may include hepatic resection, radiofrequency ablation, and more. 

Hepatic cysts are broad and diverse. The diagnosis of these conditions can guide the effective treatment of the lesions. Once diagnosed, it is vital to educate the patient on the matter and how they should approach the next steps in management.

## Case presentation

**Figure 1:**
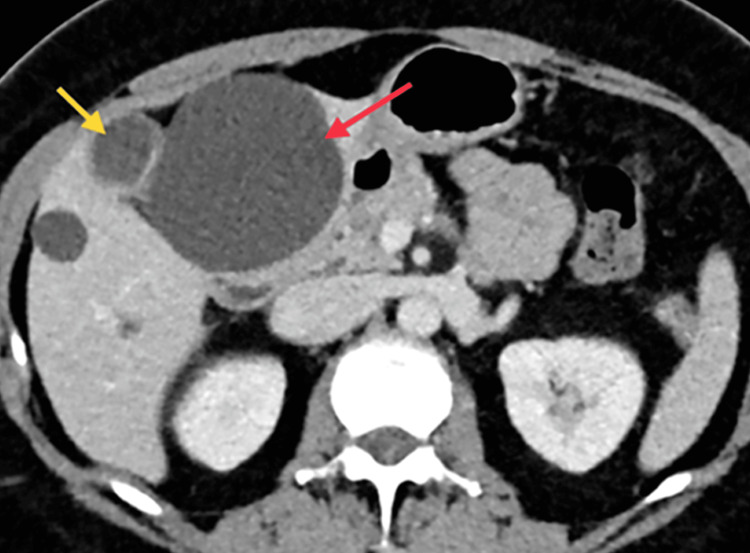
Transverse CT image cross-section of the abdomen displayed a 7.5-cm hepatic cyst (red arrow) and compressed gallbladder (yellow arrow)

**Figure 2:**
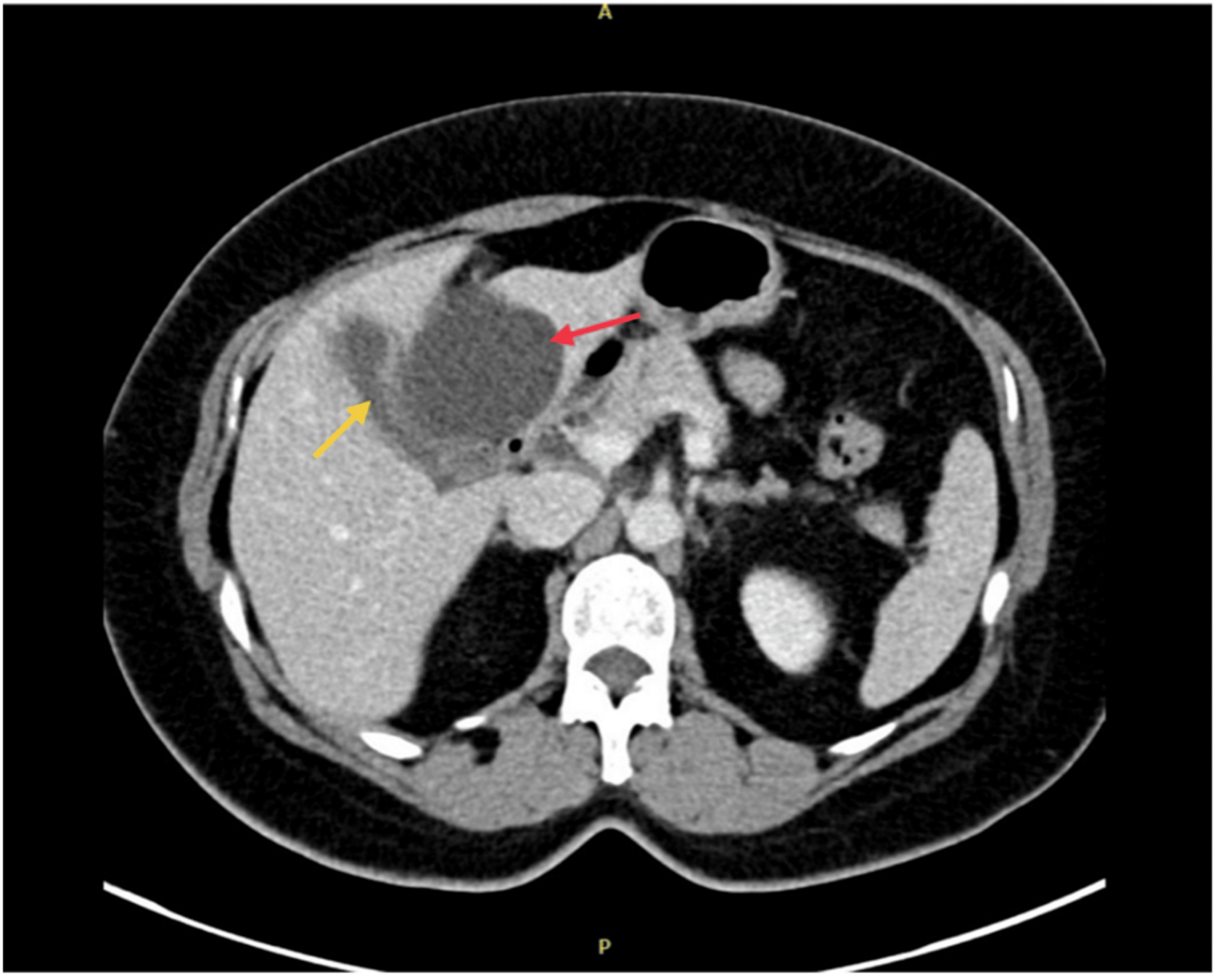
Supplemental CT image cross-section displaying visual obstruction of the gallbladder (yellow arrow) by a dominant left lobe cyst (red arrow)

We present the case of a 53-year-old woman with no significant past medical history. She presented electively with concerns of biliary colic and chronic cholecystitis. She had normal hepatic function tests and complete blood count. Initially, she underwent an ultrasound demonstrating gallstones. She also complained of vague periumbilical symptoms, which prompted a CT scan. This revealed evidence of multiple hepatic cysts, the largest in the left lobe of the liver (Figure 1). The dominant left hepatic cyst measured 7.5 cm and exerted a mass effect on the gallbladder (Figure 2). Differential diagnosis is wide but includes simple hepatic cyst, abscess, biliary cystadenoma, hamartoma, biloma, duplication cyst, and cystadenocarcinoma. Discussion occurred with the patient regarding concurrent fenestration and excision of the cyst with biopsy along with cholecystectomy. 

**Figure 3:**
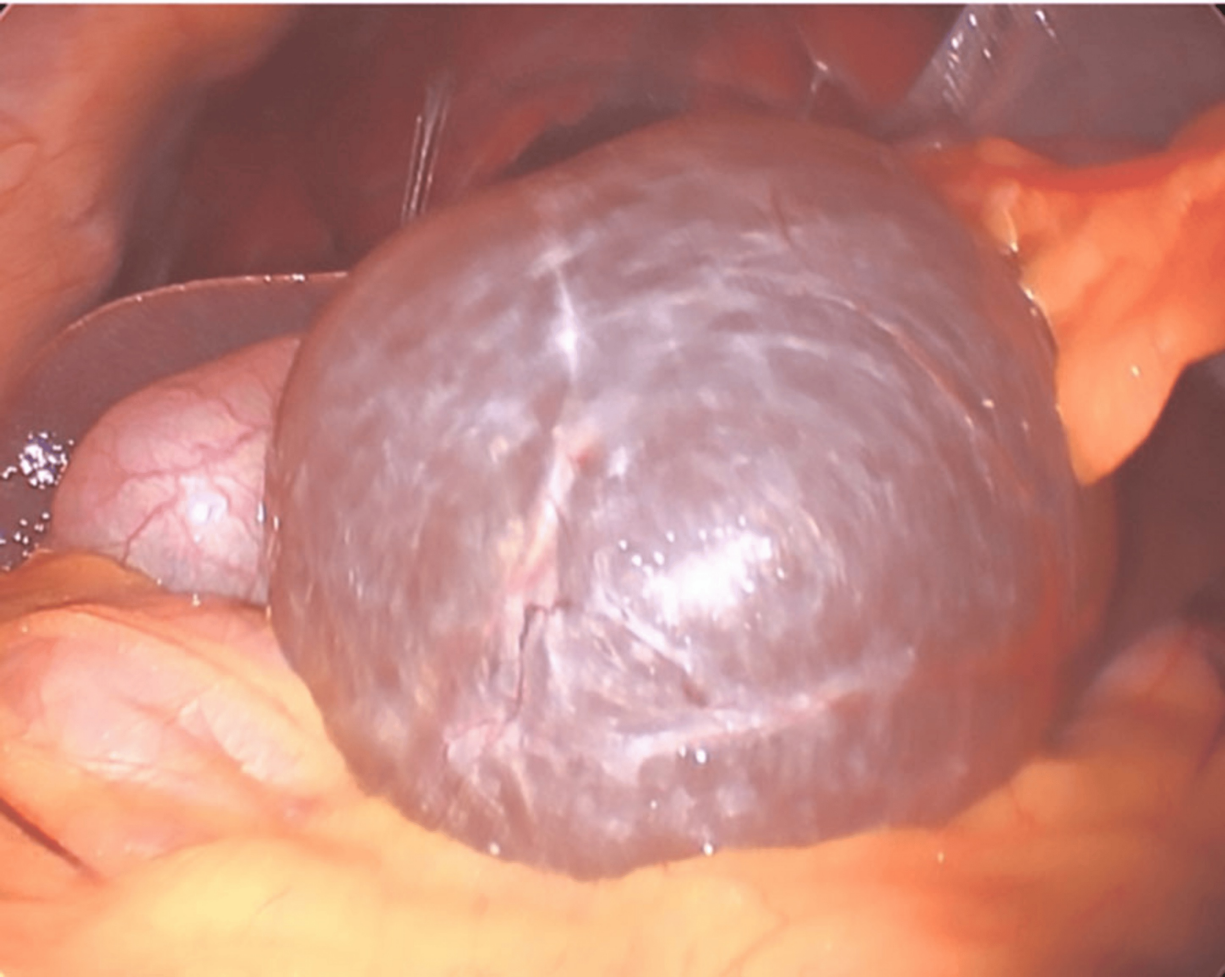
Intraoperative image during laparoscopic cholecystectomy displaying a hepatic cyst obstructing access to the gallbladder

**Figure 4:**
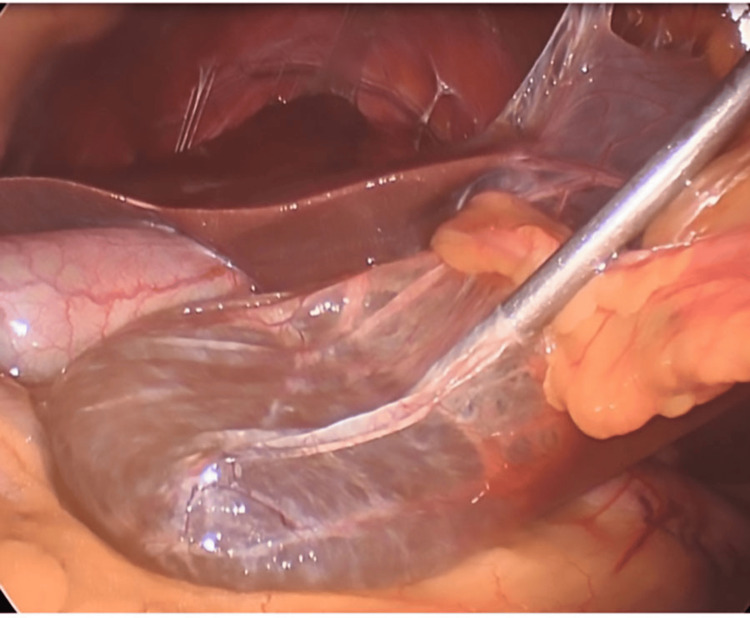
Intraoperative image displaying fenestration and decompression of the hepatic cyst

**Figure 5:**
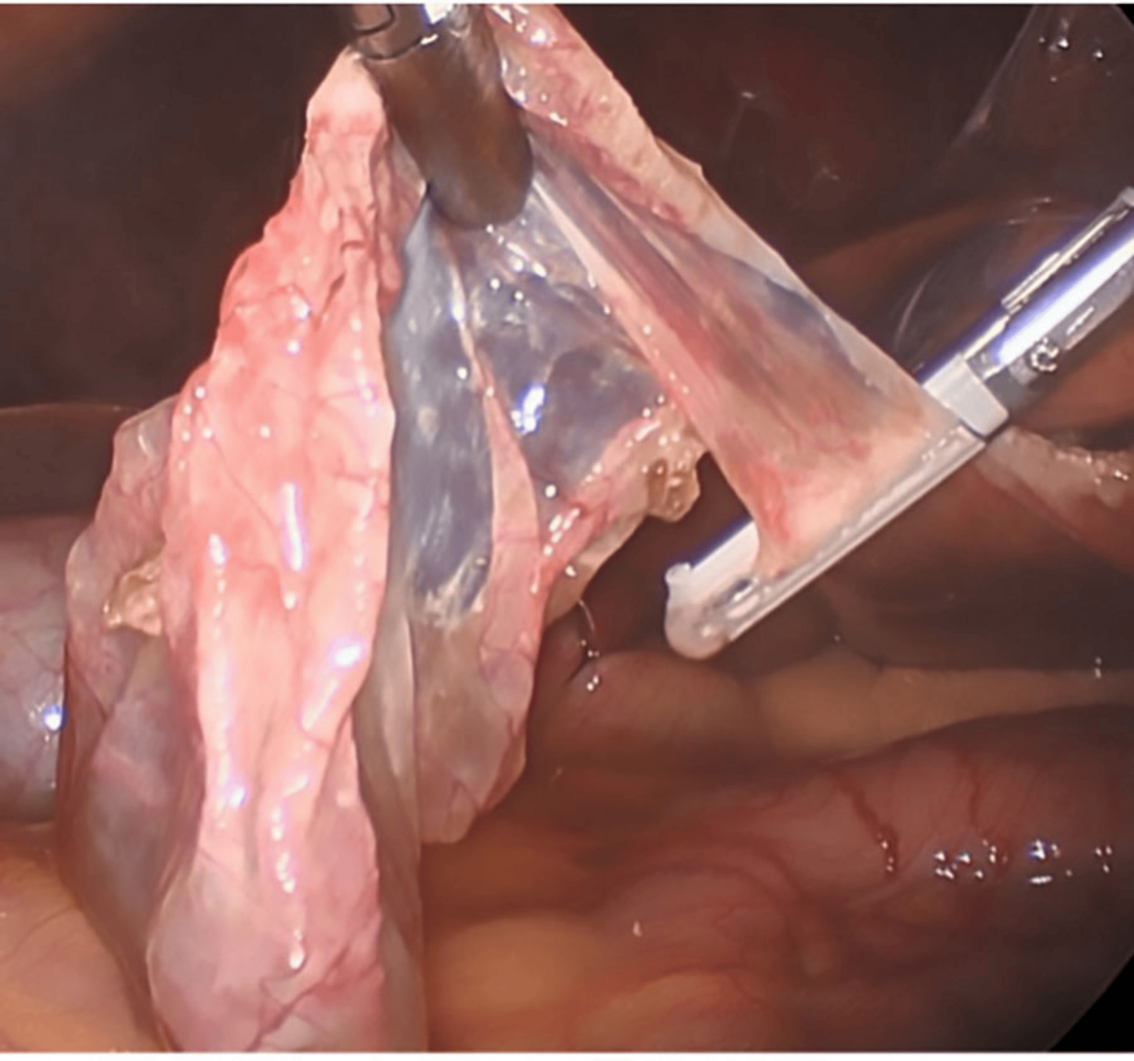
Intraoperative image displaying resection of the hepatic cyst

During surgery, laparoscopic exploration confirmed radiographic findings of a dominant inferior left lobe cyst obstructing anterior visualization of the gallbladder (Figure 3). Evacuation of the cyst fluid was commenced with blunt dissection (Figure 4), and excision of the cyst cavity was performed with bipolar cautery (Figure 5). The cavity was resected and sent for pathologic evaluation. Cholecystectomy was commenced in the standard fashion without complication. The patient had a standard postoperative course and was sent home the same day after observation in the recovery unit. 

The patient was seen in the clinic three weeks postoperatively, doing quite well with minimal pain. Pathology revealed a simple cyst and chronic cholecystitis with cholelithiasis.

## Discussion

Hepatic cysts are a condition classified from benign to malignant. Cysts may exhibit non-specific symptoms such as nausea, pain, and shortness of breath [5]. Prevalence rates for hepatic cysts in the United States are between an estimated 15% and 18% [5,6] Simple cysts are the most common type of hepatic cysts in the United States, with higher prevalence rates in females than males [5].

The underlying risk factors for hepatic cysts carry a wide differential diagnosis, ranging from infectious and inflammatory to malignant, with genetic conditions occasionally playing a role in their etiology. Polycystic liver disease (PLD) is an example of a benign genetic condition predisposing to the development of benign hepatic cysts. Diagnosis is made with ultrasound and cross-sectional imaging (CT, liver-phase MRI). Core biopsy is rarely indicated due to concern for potential free spillage of cyst contents, particularly in an infectious etiology. Due to the non-specific nature of symptoms, there is a potential for misdiagnosis and overlap with other conditions such as chronic cholecystitis. 

Once a diagnosis is determined, implementation of a treatment plan should be considered, especially if there is a concern that the cysts are causing symptoms. Treatment options such as laparoscopic surgery, fenestration, decompression, marsupialization, and excision of the cyst cavity can prove beneficial. In this setting, these methods were undertaken and the patient had an excellent postoperative outcome. Caution should be taken if the intraoperative appearance of the mass is more blood-filled or solid in nature, as these characteristics can signify a hepatic hemangioma or tumor. We resected the hepatic cyst secondary to its obstruction of the gallbladder, thereby contributing to a safe cholecystectomy. A biopsy was completed to confirm the diagnosis of a simple cyst. 

Cholecystitis, acute or chronic, overlaps in its symptoms with hepatic cysts. Gallbladder disease presents with epigastric pain and is more readily diagnosed [10]. As imaging studies can clarify the disease progression, additional conditions may also be identified. With concurrent cholecystitis and hepatic cyst discovery, plans to manage both during the index operation may be necessary.

## Conclusions

Laparoscopic cholecystectomy is a minimally invasive procedure intended for the resection of the gallbladder. Appropriate imaging studies should be completed before the determination of cholecystectomy. At times, relevant incidental findings may necessitate a change in management. When a hepatic cyst causes an impediment to the safe completion of the laparoscopic procedure, measures must be taken to concurrently manage the cyst.
